# Switching of multi-state magnetic structures via domain wall propagation triggered by spin-orbit torques

**DOI:** 10.1038/s41598-019-56714-2

**Published:** 2019-12-30

**Authors:** Shubhankar Das, Ariel Zaig, Hariharan Nhalil, Liran Avraham, Moty Schultz, Lior Klein

**Affiliations:** Department of Physics, Nano-magnetism Research Center, Institute of Nanotechnology and Advanced Materials, Bar-Ilan University, Ramat-Gan, 52900 Israel

**Keywords:** Ferromagnetism, Spintronics

## Abstract

Spin-orbit torques emerge as a promising method for manipulating magnetic configurations of spintronic devices. Here, we show that these torques can induce a magnetization reversal via domain wall propagation which may open new ways in developing novel spintronic devices and in particular in realizing high-density multi-level magnetic memory. Our devices are bi-layer heterostructures of Ni_0.8_Fe_0.2_ on top of *β*-Ta patterned in the form of two or three crossing ellipses which exhibit in the crossing area shape-induced biaxial and triaxial magnetic anisotropy, respectively. We demonstrate field-free switching between discrete remanent magnetic states of the structures by spin-orbit torques induced by flowing electrical current through one of the ellipses. We note switchings induced by the coupling between the ellipses where current flowing in one ellipse triggers a reversal in a neighboring ellipse which propagates from the center outwards. Numerical tools successfully simulate the observed coupling-induced switching using experimentally extracted parameters.

## Introduction

Magnetization switching of magnetic structures has been extensively studied in recent decades; particularly, due to its relevance to the operation of magnetic memory devices. Following the birth of spintronics and the emergence of magnetic random access memory (MRAM) as a promising non-volatile and fast memory, focus turned into the search of efficient and scalable methods to manipulate the magnetic state of one of the magnetic layers in the magnetic tunnel junction (MTJ) which constitutes a basic two-level memory bit of MRAM. Initial MRAM devices which used the Oersted fields generated by an array of non-magnetic conducting wires were soon abandoned due to lack of scalability for new MRAM devices that utilize spin transfer torques (STT) induced by the injection of spin polarized current in to the MTJ^1–5^. However, it was soon realized that this method is detrimental for the MTJ^5^, paving the way for the use of spin-orbit torques (SOTs) which utilize the effect that flowing a charge current in a heavy metal layer adjacent to a ferromagnetic layer generates a spin current flowing perpendicular to the heavy metal/ferromagnetic (HM/FM) interface into the FM layer without accompanying charge current^6–10^.

In recent years, studies of SOT-induced magnetization switching have been performed extensively on structures with uniaxial anisotropy where the magnetization is uniform^11–24^. In addition, SOTs have been used to systematically control domain wall motion for new classes of data, memory and logic devices^9,25–30^. In a recent report^31^ we showed the efficiency of SOTs in inducing switchings in structures which exhibit non-uniform magnetization and effective shape-induced bi-axial magnetic anisotropy. These structures when used as one of the magnetic layers in a MTJ may pave the way for novel multi-level MRAM with efficient write operation based on SOTs.

Here we show a new type of magnetization switching induced by SOTs. Commonly, magnetization switching induced by SOTs is studied where the entire studied magnetic device experiences the same injection of spin currents. Here, the spin current affects only a small part of the structure; however, the magnetization reversal propagates away from the region subject to spin currents until a full switching is achieved. Such type of reversal not only makes the switching of multi-level MRAM more efficient, but it also paves the way for novel spintronic devices.

We study magnetization switchings in bi-layer heterostructures of Ni_0.8_Fe_0.2_ on top of *β*-Ta patterned in the form of two or three crossing ellipses which exhibit in the crossing area shape-induced biaxial and triaxial magnetic anisotropy, respectively. We show switchings between discrete magnetic remanent states of the structures by flowing current through individual ellipses where the current flowing in the Ta layer generates torques on the Ni_0.8_Fe_0.2_ layer on top of it. Interestingly, we find that flowing current through a particular ellipse may induce magnetization switching in a neighboring ellipse. Such a switching occurs due to the magnetic coupling between the ellipses which share a crossing area. Namely, a change in the magnetization orientation in the crossing area initiates a magnetization reversal via domain wall propagation in the entire neighboring ellipse. To quantify the strength of the different types of spin orbit torques contributing to the switchings, we measure high harmonic planar Hall effect resistance of Hall bar patterns fabricated from the same bi-layer heterostructures, and the results are used to simulate the observed switching. The current required for switching when it flows through a single ellipse is smaller than the current needed when it flows under the entire structure which is of high importance for applications such as multi-state MRAM. Furthermore, such type of reversal increases the possibilities of manipulating the magnetic structures.

## Results

### The studied devices

**Figure 1:**
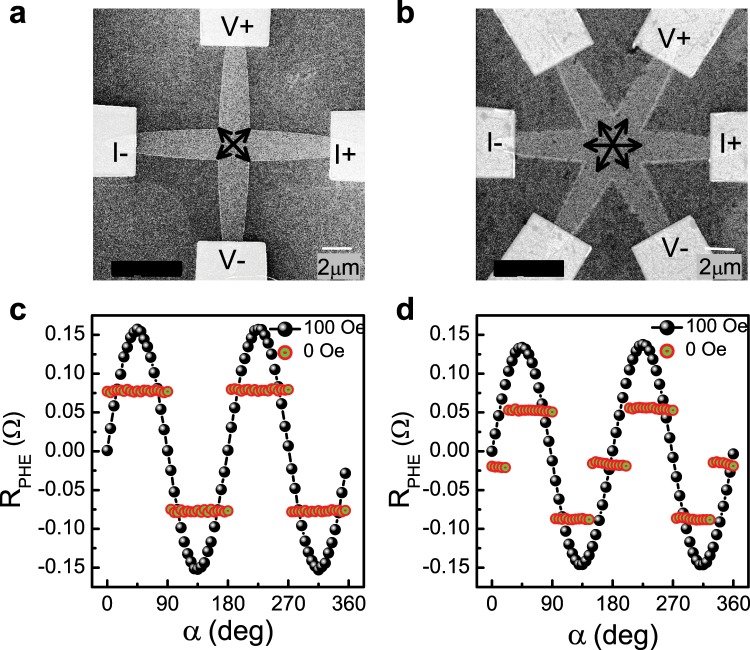
The structure and characterization of the devices. (**a**,**b**) Scanning electron microscopy images of Devices 1 and 2, respectively. Current and voltage pads are indicated. The direction of easy axes in the overlap area are shown by arrows. (**c**,**d**) R_PHE_ measured with a field of 100 Oe (black) and after the field is switched off (red) as a function of the angle *α* between the field and the current for Devices 1 and 2, respectively.

Two types of devices are studied. Device 1 consists of two crossing ellipses (Fig. 1(a)) and Device 2 consists of three crossing ellipses (Fig. 1(b)), where the principal axes of each ellipse are 2 and 16 *μm* long. The current contact pads are attached to the edges of the ellipses. Taking into account the thickness and resistivities of the different layers, we estimate that a current of 2 mA would generate a current density of 0.75 × 10^7^ A/cm^2^ in the Ta layer. In our calculations, we neglect the current flowing in the arms across which the voltage is measured. The current through the Ta-layer generates SOTs on the FM layer. We note that the capping layer of Ti is effectively thinner due to oxidation and in addition its spin-Hall angle is relatively small. Consequently, the contribution of the Ti layer to SOTs on the permalloy layer is negligible.

The magnetization of the devices is monitored by measuring the planar Hall effect (PHE) resistance, R_PHE_. For magnetic conductors where the crystal symmetry is averaged out (as in polycrystalline samples) or its effect on transport is negligible, R_PHE_ is given by R_PHE_ = $\frac{1}{2}\Delta {\rm{R}}\,\sin \,2\theta$, where ΔR is the anisotropic magnetoresistance amplitude and *θ* is the angle between current and magnetization^32^. Figure 1(c,d) present R_PHE_ for Devices 1 and 2 as a function of the angle between the current and the field (*α*) with a saturating field (100 Oe) and after the field is switched off, for each angle *α*. The sin2*θ* dependence when the saturating field is on (and the magnetization is parallel to the applied field) indicates that R_PHE_ reflects the magnetization in the crossing area of the devices. The observed plateaus obtained with the field switched off reflect the symmetry of the shape induced magnetic anisotropy. Device 1 shows four plateaus indicating biaxial anisotropy with easy axes pointing in between the long axes of the ellipses, whereas Device 2 exhibits six plateaus indicating triaxial anisotropy, with easy axes parallel to the long axes of the ellipses. In both of the devices, magnetization in the arms away from the overlap area is parallel to the long axes. A comprehensive study of magnetic behavior of structures such as Device 1 and Device 2 were presented in ref. ^31,33–35^ respectively.

### SOTs-induced switching

**Figure 2:**
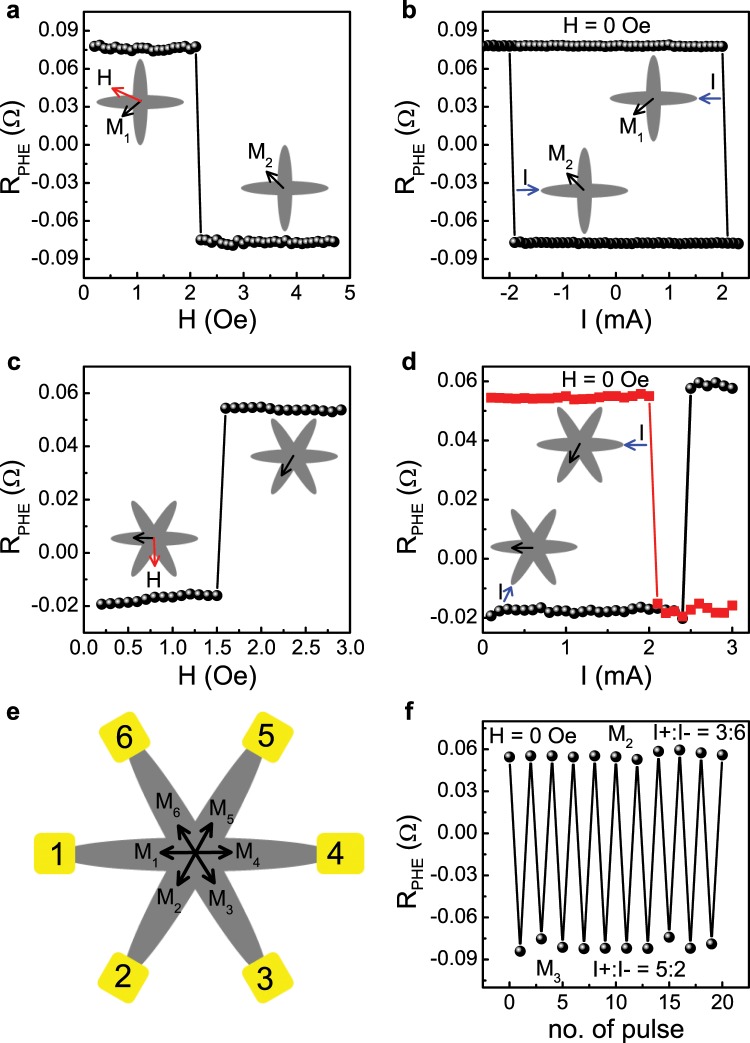
SOT-induced switching of the devices. (**a**) R_PHE_ vs H for Device 1. The device is prepared with magnetization in M_1_ state and the field is applied at *α* = 330 deg. The data are taken for each field step after the field is switched off. The magnetization states and the field direction are indicated. (**b**) R_PHE_ vs I for Device 1. The device is prepared either with magnetization in M_1_ state or with magnetization in M_2_ state. The data are taken for each current step with a probing current of 50 *μ*A. The magnetization states and the current direction are indicated. (**c**,**d**) R_PHE_ vs H and R_PHE_ vs I, respectively, for Device 2. The m**e**asurements are the same as in (**a,b**). (**e**) A schematic illustration of Device 2 indicating the remanent states in the overlap area and the numbers denoting the contact pads. (**f**) Current pulses of 2.7 mA along 3–6 and 5–2 arms are applied alternatively to induce successive switchings between states M_2_ and M_3_.

We now turn to explore the switching behavior of the devices. Figure 2(a,b) show field-induced and field-free current induced switchings, respectively, between two remanent states of Device 1 and Fig. 2(c,d) depict the same for Device 2. Figure 2(e) presents a schematic picture of Device 2 and Fig. 2(f) demonstrates successive field-free current-induced switchings between two remanent states of Device 2 which reflect the reproducibility of the switchings.

The current-induced switchings measurements are performed by driving current pulses of varying amplitude through one of the arms followed by measuring R_PHE_ with a small probing current. We note that the switching current (I_SW_) of Device 1 and 2 of ~2 mA corresponds to current density (J_SW_) of 0.75 × 10^7^ A/cm^2^ through the Ta-layer, which is almost 2 times smaller than J_SW_ needed to switch a single ellipse^31^, namely, the switching energetically more efficient (see supplementary materials for details). The spin Hall angle (*θ*_SH_) in such heterostructures is calculated and presented in details in our previous work ref. ^31^which yields *θ*_SH_ = 0.096. Here we note that while the reversible switching is performed in Device 1 by driving current in one arm, for Device 2 current is driven through different arms. Interestingly, in the both devices we note switchings where current flowing along one ellipse induces switching in a neighboring ellipse. Additional current-induced switchings of Device 2 are shown in Fig. S3 in the supplementary material. Switching current values and directions for all initial remanent states of Device 2 are listed in Table I in thesupplementary material. We note that M_1_ state is switched to M_2_ state by flowing current in the 2–5 arm, whereas according to the Oersted field scenario a switch to M_3_ state would be expected (see supplementary materials for details).

### Harmonic hall measurements

To elucidate the mechanisms responsible for the observed switchings, we have performed harmonic transverse voltage measurements^36^ which are employed to determine the current-induced SOTs in HM/FM heterostructure with out-of-plane^37–39^ or in-plane^40–42^ magnetic anisotropies. For structures with in-plane magnetization, if the external magnetic field is strong enough to rotate the magnetization coherently, the second harmonic transverse resistance (R^2*ω*^) is given by^40–42^

1 $$\begin{array}{rcl}{{\rm{R}}}^{2\omega } & = & \frac{{{\rm{H}}}_{{\rm{FL}}}{{\rm{R}}}_{{\rm{P}}}}{{{\rm{H}}}_{{\rm{K}}({\rm{i}})}-{\rm{H}}}\,\cos \,\alpha \,\cos \,2\alpha \\  &  & +\,(\frac{1}{2}\frac{{{\rm{H}}}_{{\rm{AD}}}{{\rm{R}}}_{{\rm{A}}}}{{{\rm{H}}}_{{\rm{K}}({\rm{o}})}-{\rm{H}}}+{\rm{A}}\beta \nabla {\rm{T}}+{\rm{N}}\beta {\rm{H}}\nabla {\rm{T}})\cos \,\alpha \end{array}$$

where H_FL_ and H_AD_ are the field-like (FL) and anti-damping (AD) effective field, respectively; H_K(i)_ and H _K(o)_ are the in-plane and out-of-plane anisotropy field, respectively; R_P_, R_A_, A and N are the coefficients of PHE, anomalous Hall effect (AHE), anomalous Nernst effect (ANE) and ordinary Nernst effect (ONE), respectively; *β* is a geometrical factor and ▽T is the temperature gradient. The cos *α*cos2*α* term aries from the FL torque, cos *α* contribution aries from AD torque, ANE and ONE which correspond to the first, second and third terms in the parenthesis, respectively.

**Figure 3:**
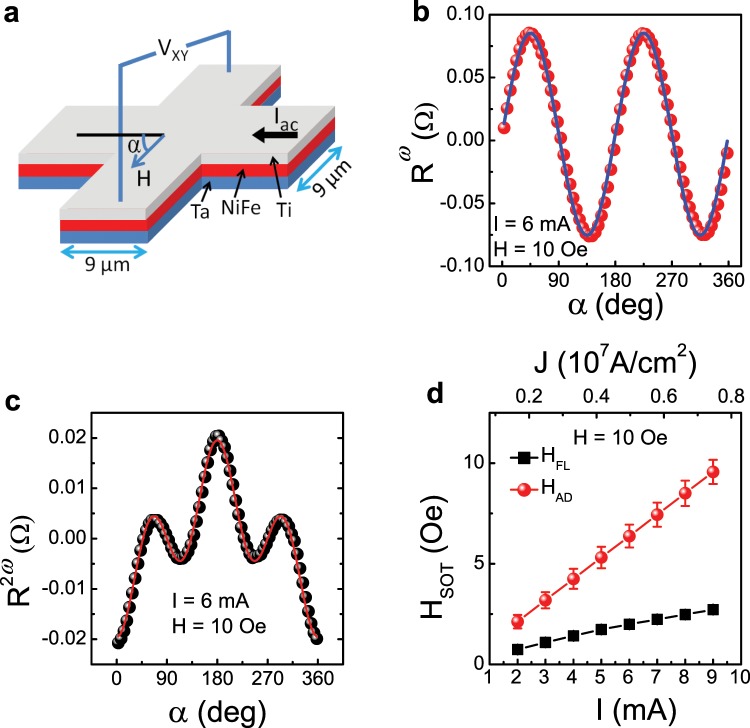
Determination of SOT effective fields. (**a**) A schematic illustration of the Hall geometry used to measure higher order harmonic measurements. (**b**) R^*ω*^ vs *α* with a current of 6 mA and a field of 10 Oe. (**c**) R^2*ω*^ as a function of the magnetic field direction is measured with the same current and magnetic field. The red line is a fit using Eq. 1. (**d**) The current dependence of SOTs effective field value, extracted from the fitting of (**c**). A small offset due to the slight misalignment of the Hall bar is subtracted from the data.

The equation cannot be readily applied to our devices since in our devices the magnetization is non-uniform and the anisotropy is of high order. Therefore, to extract SOT effective field (H_SOT_), we have measured a Hall bar, patterned using the same heterostructure used to fabricate Devices 1 and 2, with a crossing area of 9 × 9 *μ*m^2^ for which the shape anisotropy is negligible (schematic illustration is shown in Fig. 3(a)). Figure 3(b) shows the first harmonic transverse resistance (R^*ω*^) for the patterned Hall bar as a function of field direction measured with 6 mA (J = 0.5 × 10^7^ A/cm^2^) and 10 Oe. We note that the data fit very well with the equation R^*ω*^ = R_P_sin2*α*, which indicates that the magnetization rotates coherently with the field. The *α*-dependence of R^2*ω*^ is shown in Fig. 3(c) and the red line is the fit using Eq. 1. Using H_K(i)_ = 1.9 ± 0.04 Oe, H_K(o)_ = 5383 ± 2.3 Oe, R_P_ = 0.08 Ω and R_A_ = 1.10 Ω (see supplementary material for details), the fitting in Fig. 3 yields H_FL_ = 2 ± 0.03 Oe and H_AD_ = 6.4 ± 0.82 Oe.

Figure 3(d) shows H_AD_ and H_FL_ as a function of current. H_AD_ has been calculated after subtracting the Nernst effect contribution to R^2*ω*^ (see Fig. S7 of supplementary material for details). We note that the corresponding field-free J_SW_ (0.75 × 10^7^ A/cm^2^) for the devices, yields H_FL_ and H_AD_ of 2.6 ± 0.04 Oe and 9.9 ± 0.95 Oe, respectively. The results indicate the anti-damping torque plays a role in the observed switching in such heterostructure (see switching mechanism section of supplementary materials for details). The *θ*_SH_ obtained from harmonic Hall measurements can be written as *θ*_SH_ = $\frac{2e}{\hslash }\frac{{{\rm{H}}}_{{\rm{AD}}}{{\rm{M}}}_{{\rm{S}}}{{\rm{d}}}_{{\rm{FM}}}}{{{\rm{J}}}_{0}^{{\rm{Ta}}}}$, where M_S_ is the saturation magnetization, d_FM_ is thickness of ferromagnetic layer and ${{\rm{J}}\,}_{0}^{{\rm{Ta}}}$ is the current density through Ta. Now using H_AD_ = 9.9 Oe, M _S_ = 7.95 × 10^5^ A/m^2^, d_FM_ = 2 nm and ${{\rm{J}}\,}_{0}^{{\rm{Ta}}}$ = 0.75 × 10^7^ A/cm^2^, *θ*_SH_ yields 0.064, whereas from the dc switching experiment the obtained *θ*_SH_ is 0.096. The reduction of calculated *θ*_SH_ from harmonic Hall measurements can be understood from the current shunting in the voltage pick up bar due to the high aspect ratio of the voltage pick up lines to the current channel width, i.e. 1:1, in our Hall bar structures^43^.

### Micro-magnetic simulations of switching

**Figure 4:**
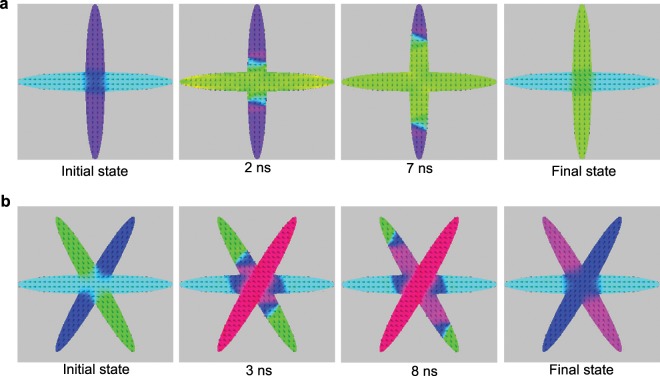
Micro-magnetic simulation of switching. (**a**) Micro-magnetic simulated magnetization switching in Device 1. The transition is obtained with a simulated spin-current pulse of amplitude 3 × 10^6^ A/cm^2^ and duration 13 ns in the horizontal arm. (**b**) The Switching between two remanent states are also performed in Device 2 with a simulated spin-current pulse of amplitude 7 × 10^6^ A/cm^2^ and duration 13 ns in the blue arm (2–5 arm). The arrows indicate the magnetization direction. The color map is shown in Fig. S1 of supplementary materials.

To further explore the switching dynamics, we have carried out micro-magnetic simulation (MuMax^3^ ref. ^44^) considering both anti-damping and field-like torques. We consider the ratio of field-like and anti-damping torque term as extracted from the above harmonic Hall measurements. Figure 4(a) shows the time evolution of switching in Device 1 by driving a spin-polarized current pulse with magnitude and duration of 3 × 10^6^ A/cm^2^ and 13 ns, respectively, in the horizontal arm. The final state is obtained by relaxing the structure after flowing the pulse. The relaxation time depends on structure dimension and the particular magnetic configuration at the time the pulse stopped. The ellipse dimensions are 2.048 × 16.384 *μ*m^2^ of thickness 2 nm. The relaxation time for this simulated structures after the magnetization reached its steady state with the current is 5 ns. Here we note that while the magnetization of the horizontal arms in both of the remanent states (M_1_ and M_2_) point towards the same direction, the magnetization direction in the vertical arms reverses. The detailed simulated time-evolution of the switching is shown in Fig. S1 in the supplementary material which demonstrates that the switching is accomplished by domain wall propagation in the crossing arm. We note that irrespective of sample dimensions, the response time to spin torque is less than 0.2 ns and the time needed for the domain wall to propagate to the edge of the ellipse depends on the size of the structure and domain wall velocity. Similar simulations were performed for structures consisting of three crossing ellipses. Figure 4(b) shows the time evolution of the switching, where a spin-polarized current pulse with magnitude and duration of 7 × 10^6^ A/cm^2^ and 13 ns, respectively, in the 2–5 (blue) arm was used. The detailed simulated time-evolution of the switching triggered by domain wall propagation is shown in Fig. S2 in the supplementary material. Here we clearly see that the current pulse affects the magnetization in the overlap area and that the reversal front propagates in the neighboring arm from the center to outwards.

## Discussion

The nature of SOTs which are induced on a magnetic structure by current flowing in an adjacent heavy metal layer makes it easier to apply such torques locally on selected parts of the magnetic structure which provides a powerful tool in manipulating the magnetization in various spintronic devices, including the possibility to realize magnetic states which are unaccessible when the entire magnetic structure is subject to such torques. Here we show field-free switching, via domain wall propagation in the neighboring ellipse, between the remanent states of a multi-level magnetic structure. The trend of switching is that if the direction of the current is opposite (along) to the direction of the magnetization in the ellipse arm, the magnetization rotation in the overlap area would be anticlockwise (clockwise). This demonstration is important for realizing a novel multi-level MRAM which may allow a significant increase in memory density (see supplementary materials for details); however, the results may also pave the way for other types of spintronic devices, where such-type of switching may be used for transferring information in a more complicated device. We also note that memory with multiple states is relevant to the growing interest in developing memristors^45^ and magnetic analogs to neural computation^46^. Furthermore, the presented results open new opportunities for studying the effect of SOTs in unexplored conditions where the magnetization of the studied structures is nonuniform and exhibits high order magnetic anisotropy.

## Methods

The heterostructure, consisting of *β*-Ta(5 nm)/Ni_0.8_Fe_0.2_(2 nm)/Ti(3 nm), are deposited by ion-beam sputtering on thermally oxidized Si-wafer as described elsewhere^47^. The resistivity of Ta is ~210 *μ*Ω-cm which indicates the *β*-phase. E-beam lithography followed by Ar-ion etching are performed to pattern the crossing ellipses. Subsequently, e-beam lithography and Au-sputtering are carried out for contact pads deposition. Finally, the devices are wire bonded and measured in a home-made system consisting of a Helmholtz coil and a sample rotator with angular precision of 0.03^0^. Four probe geometry is used to measure R_PHE_ (=V_XY_/I), which eliminates the contact resistance. All measurements are performed at room temperature.

## Supplementary information

Supplementary information
