# Supplementary material for: Switching of multi-state magnetic structures via domain wall propagation triggered by spin-orbit torques
Source: Sci Rep. 2019 Dec 30;9:20368. doi: 10.1038/s41598-019-56714-2 (PMC6937281; doi:10.1038/s41598-019-56714-2)
Supplement: Supplementary file 1 — Supplementary information [file 41598_2019_56714_MOESM1_ESM.pdf]

Supplementary - Switching of multi-state magnetic structures via domain wall propagation triggered by spin-orbit torques

Shubhankar Das, Ariel Zaig, Hariharan Nhalil, Liran Avraham, Moty Schultz, and Lior Klein

Department of Physics, Nano-magnetism Research Center,

Institute of Nanotechnology and Advanced Materials,

Bar-Ilan University, Ramat-Gan 52900, Israel

(Dated: November 14, 2019)

Time-evolution of switchings in Devices 1 and 2

Fig. S1 shows the micromagnetic simulation (MuMax^3 Ref. [1]) of time evolution of switching of Device 1 by flowing a spin-polarized current pulse of amplitude 3×10^6 A/cm^2 and duration of 13 ns in the horizontal arm. We simulate a ellipse of dimension 2.048×16.384 μm^2 and thickness of 2 nm. We consider the Gilbert damping co-efficient of 0.01, saturation magnetization (M_S) = 8×10^5 A/m and exchange stiffness (A_{ex}) = 1.3×10^{-11} J/m^2, characteristics of permalloy. The initial state is obtained by applying a field along M_1 and allowing the system to relax after turning off the field. After the current pulse is driven in the horizontal arm, the final state is obtained by relaxing the structure. The propagation of magnetization along the vertical arm is clearly observed.

Fig. S2 shows the micro-magnetic simulation of switching of Device 2 by flowing a spin-polarized current pulse of amplitude 7×10^6 A/cm^2 and duration of 13 ns in the 2-5 arm (please see the main text for numbering). The ellipse dimension are 2.048×16.384 μm^2 and thickness of 2 nm. Here also, the propagation of magnetization along neighboring (3-6) arm is clearly observed.

The magnetization dynamics of a magnetic region with uniform magnetization $\vec{m}$ subject to external fields and spin orbit torques associated with current flowing in an adjacent HM layer is commonly described by the Landau-Lifshitz-Gilbert-Slonczewski (LLGS) equation [2, 3];

$$\frac{d\vec{m}}{dt} = -\gamma\vec{m}\times\vec{H_{eff}} + \alpha\vec{m}\times\frac{d\vec{m}}{dt} + H_{FL}\vec{m}\times\vec{\sigma} + H_{AD}\vec{m}\times(\vec{m}\times\vec{\sigma}) \tag{S1}$$

where the first term corresponds to the magnetic precession due to an effective magnetic field $\vec{H_{eff}}$ (which includes external field, anisotropy field and Oersted field) and γ is the gyromagnetic ratio, the second term corresponds to the magnetic damping and α is the Gilbert damping coefficient, the third term is a field like term where $\vec{\sigma}$ is the normalized spin polarization direction of the spin current, and the fourth term is the anti-damping term.

The Mumax^3 simulation is based on the numerical solution of LLGS equation. Mumax^3 solves the time and space dependent magnetization evolution in nano to micro scale magnets using a finite-difference discretization. The simulation derives the evolution of the reduced magnetization $\vec{m}(\vec{r},t)$. Mumax^3 refers to the time derivative of $\vec{m}$ as the torque $\vec{\tau}$ [1];

$$\vec{\tau} = \frac{\partial\vec{m}}{\partial\vec{t}} \tag{S2}$$

$\vec{\tau}$ has three possible contributions: $\vec{\tau}_{LL}$ Landau-Lifshitz torque,$\vec{\tau}_{ZL}$ Zhang-Li spin-transfer torque and $\vec{\tau}_{SL}$ Slonczewski spin-transfer torque. We use Slonczewski spin-transfer torque formalism to perform the simulations. Slonczewski spin-transfer torque term can be written as [1];

$$\overrightarrow{\tau}_{SL} = \beta\frac{\varepsilon - \lambda\varepsilon'}{1+\lambda^2}\left(\overrightarrow{m} \times \left(\overrightarrow{\sigma} \times \overrightarrow{m}\right)\right) - \beta\frac{\varepsilon' - \lambda\varepsilon}{1+\lambda^2}\overrightarrow{m} \times \overrightarrow{\sigma} \tag{S3}$$

where $\beta = \frac{j_Z\hbar}{M_{sat}ed}$, $\varepsilon = \frac{P(\overrightarrow{r},t)\Lambda^2}{(\Lambda^2+1)+(\Lambda^2-1)(\overrightarrow{m}\cdot\overrightarrow{\sigma})}$, j_Z is the current density along the z axis, d is the free layer thickness, $\vec{\sigma}$ is the spin polarization of spin current, P the spin polarization, the Slonczewski Λ parameter characterizes the spacer layer and ε' is the secondary spin-torque parameter. The magnitudes of the anti-damping and field-like terms contributions are decided by varying Λ and ε'. We have used P = 0.5, Λ = 0.5 and ε' = 0.471 in the simulation.

SOT induced reversible switchings in Device 2

The field-free all electrical reversible switchings between M_2 and M_3, M_3 and M_4, M_4 and M_5, M_6 and M_1 are shown in Fig. S3(a), (b), (c) and (d), respectively. Table I shows the switching current (magnitude and direction) for all initial remanent states.

Energy efficient switching: As mentioned, the current density needed to switch the single ellipse structure is $\sim$1.4 $\times$ 10^7 A/cm^2 [4], whereas for two- and three-crossing ellipses it is $\sim$0.75 $\times$ 10^7 A/cm^2. The power dissipation can be written as W = I^2R = $J^2\rho$lwt, where R is the resistance across current pads, I and J are the switching current magnitude and the current density, respectively, ρ is the resistivity of Ta layer, l is the distance between the current pads, w and t are the width and thickness of the current channel, respectively. For the device consisting of a single ellipse presented in Ref. [4], ρ = 210 $\mu\Omega$-cm, l = 80 μm, w = 16 μm and t = 5 nm, the energy dissipation for switching is W = 2.6 $\times$ 10^{-1} Watt. For the devices consisting of two- or three-crossing ellipses, ρ = 210 $\mu\Omega$-cm, l = 14 μm, w = 2 μm and t = 5 nm, the energy dissipation for switching is W = 1.6 $\times$ 10^{-3} Watt.

Switching mechanism

The switching from initial state (M_1) to final state (M_2) in absence of any external field is performed by driving a current in 2-5 arm (Fig. 2(d) of main text). Due to the current an Oersted field (H_{Oe}) would generate at an angle $\alpha = 150$ deg (shown in Fig. S4(a)). We calculated the current density of 0.75×10^7 in Ta-layer yields H_{Oe} of $\sim$2.3 Oe. Now, we set the same initial state and apply an external field along $\alpha = 150$ deg keeping the probing current very low (50 μA), which yields the switching to M_3 state (Fig. S4(b)), instead of M_2 state. This result strongly indicates that the dominant contribution of switching comes from mechanism other than Oersted field, though we can not rule out the contribution from Oersted field.

Fan et al. [5] have shown by considering magnetization orientation due to spin-orbit interaction as perturbation to magnetization and using second order approximation that in-plane magnetization rotation may be realized with the assistance of the anti-damping torque. Fukami et al. [6] demonstrated with macro-spin simulations using Slonczewski-like SOT pulse that a misalignment as small as one degree between the current and the in-plane magnetization may also induce such a magnetization rotation.

The figures of the micro-magnetic simulations (mumax^3) we presented show the in-plane component of the magnetization; however, an out of plane component of magnetization does exist. Fig. S5 shows the normalized z-component of the magnetization (m_Z), where $\sqrt{m_x^2 + m_y^2 + m_z^2} = 1$, for different intermediate states during the switching. We can clearly see that there is no visible m_Z component in the initial and final states (Fig. S5(a) and (d)), whereas the intermediate states (Fig S5(b) and (c)) show a significant m_Z component which is induced in the overlap area and then propagates outwards in the switching ellipse. As the m_Z component can only be induced by anti-damping torque, the figures indicate that the anti-damping torque plays a role in the observed switching.

Anisotropy field determination

To calculate the in-plane anisotropy field ($H_{K(i)}$) the change in planar Hall resistance (R_{PHE}) is measured as a function of field applied perpendicular to the induced anisotropy direction (IAD) during growth. Prior to that, the Hall bar is fully magnetized along IAD, which is in the direction of one of the arm. By using $R_{PHE} = \frac{1}{2}\Delta R \sin 2\theta$, where ΔR is the anisotropic magnetoresistance amplitude and θ is the angle between the magnetization and current direction, the change in θ as a function of field is calculated, which is shown in Fig. S6(a). In the small field limit where the magnetization rotation is coherent with field, the change in magnetization angle can be expressed as $\Delta\theta \sim H_{\perp}/H_{K(i)}$ [4, 7]. So, from the slope of $\Delta\theta$ vs $H_{\perp}$ curve, the $H_{K(i)}$ is determined as 1.9 $\pm$ 0.04 Oe.

Fig. S6(b) shows the change in the anomalous Hall resistance (R_{AHE}) with out-of-plane field. The out-of-plane anisotropy field ($H_{K(o)}$) is determined from the crossing point of the extrapolated linear fit at low field and high field region, which yields = 5383 $\pm$ 2.3 Oe.

Harmonic Hall measurements

We have calculated the cosα contribution to second harmonic Hall resistance ($R^{2\omega}$) [8], which includes both contributions from anti-damping (AD) torque and Nernst effect(NE). The cosα contribution as a function of current is shown in Fig. S7 (solid circles). The AD torque contribution to $R^{2\omega}$ is expected to be linear with current, whereas NE contribution to $R^{2\omega}$ varies quadratically with current as $\nabla T \sim T \sim I^2R$ [8]. The black solid line in Fig. S7 is a fit using $AI + BI^2$, where A and B are fitting parameters. Using these parameters, the AD torque and NE contributions to $R^{2\omega}$ are extracted and plotted as a function of current in Fig. S7. Using the value of AD torque contribution to $R^{2\omega}$, H_{AD} has been calculated and shown as a function of current in Fig. 3(d) of main text.

Multi-state MRAM device

We propose that structures consisting of multiple crossing ellipses can be used to fabricate novel multi-level MRAM with increased memory density; namely, we can use such structures as the ferromagnetic layers of magnetic tunnel junction (MTJ). The MTJ resistance depends on the relative orientation of magnetization of the two layers; $R_{MTJ}(\beta) = R_{AV}$ - 1/2 $\Delta R \cos\beta$,

where β is the angle between the magnetization of the two layers, R_{AV} = (R_P + R_{AP})/2, R_P and R_{AP} are the MTJ resistances when magnetization of the two layers are parallel and anti-parallel, respectively and ΔR = (R_{AP} - R_P). We have performed numerical simulation considering two and three crossing ellipses as ferromagnetic layers of MTJ which yields 12 distinct states per MTJ (see Fig. S8). ΔR_{MOD} is defined as $\Delta R/24$ (as $4\times6 = 24$ is the maximum number of states possible) and ΔR_{MIN} is the minimum resistance difference between two neighboring MTJ states.

We further fabricated devices consisting of four and five crossing ellipses which show in the overlap area quadro-axial and penta-axial magnetic anisotropy, respectively. Fig. S9(a) and (b) show the SEM images of the devices, respectively. Fig. S9(c) and (d) show R_{PHE} as a function of field direction and for each field direction the data are taken at 100 Oe and after the field is switched off. The eight and ten plateaus, seen in Fig. S9(c) and (d) respectively, correspond to eight and ten remanent states of four and five crossing ellipses, respectively. We further perform simulation considering four and five crossing ellipses as the ferromagnetic layers of MTJ which yields 70 distinct states per MTJ (see Fig. S9(f)). Here ΔR_{MOD} is defined as $\Delta R/80$ (as $8\times10 = 80$ is the maximum number of states possible).

Obtaining such a large number of states per MTJ cell in addition to further decreasing the size of the structures may pave the way for multi-level MRAM with increased memory density. We also note that memory with multiple states is relevant to the growing interest in developing memristors [9] and magnetic analogs to neural computation [10].

References

[1] Vansteenkiste A. et al. The design and verification of MuMax3. *AIP Adv.* **4,** 107133 (2014).

[2] Slonczewski, J. C. Current-driven excitation of magnetic multilayers. *J. Magn. Magn. Mater.* **159,** L1-L7 (1996).

[3] Slonczcwski, J. C. Currents and torques in metallic magnetic multilayers. *J. Magn. Magn. Mater.* **247,** 324-338 (2002).

[4] Das, S. et al. Magnetization switching of multistate magnetic structures with current-induced torques. *Sci. Rep.* **8,** 15160 (2018).

[5] Fan, X. et al. Observation of the nonlocal spin-orbital effective field. *Nat. Commun.* **4,** 1799 (2013).

[6] Fukami, S., Anekawa, T., Zhang, C. & Ohno, H. A spinorbit torque switching scheme with collinear magnetic easy axis and current configuration. *Nat. Nanotechnol.* **11,** 621-626 (2016).

[7] Mor, V. et al. Planar Hall effect sensors with shape-induced effective single domain behavior. *J. App. Phys.* **111,** 07E519 (2012).

[8] Avci, C. O. et al. Interplay of spin-orbit torque and thermoelectric effects in ferromagnet/normal-metal bilayers. *Phys. Rev. B* **90,** 224427 (2014).

[9] Wang, X., Chen, Y., Xi, H., Li, H. & Dimitrov D. Spintronic Memristor Through Spin-Torque-Induced Magnetization Motion. *IEEE Electron Device Lett.* **30,** 294-297 (2009).

[10] Lequeux, S. et al. A magnetic synapse: multilevel spin-torque memristor with perpendicular anisotropy. *Sci. Rep.* **6,** 31510 (2016).

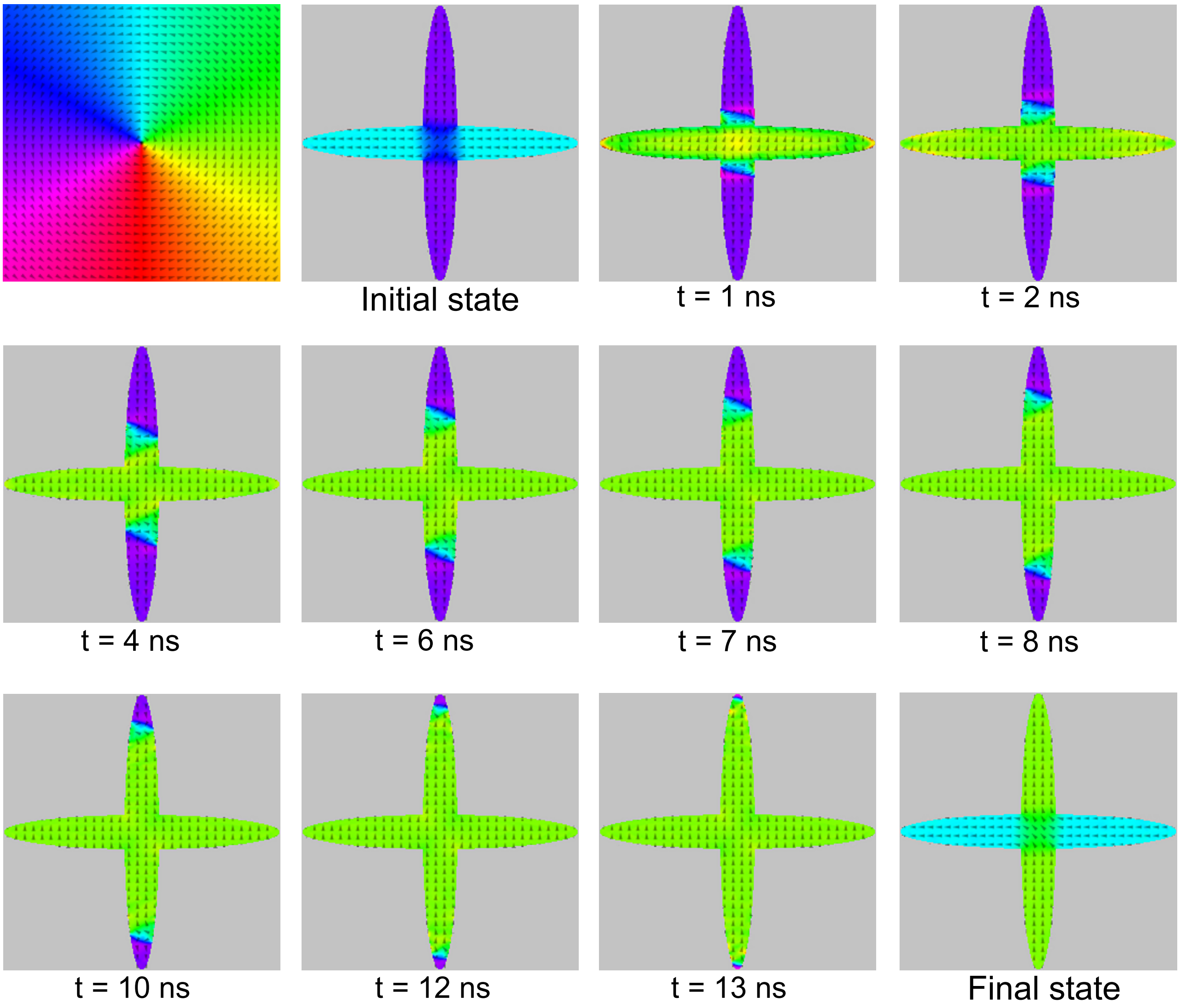

FIG. S1. **Micro-magnetic simulation of switching in Device 1.** Micro-magnetic simulation of a switching from M_1 to M_2 state in Device 1 induced by flowing a spin-polarized current pulse of amplitude 3×10^6 A/cm^2 and duration of 13 ns in the horizontal arm, perpendicular to the current direction. The top left shows the color map and the arrows indicate the magnetization direction.

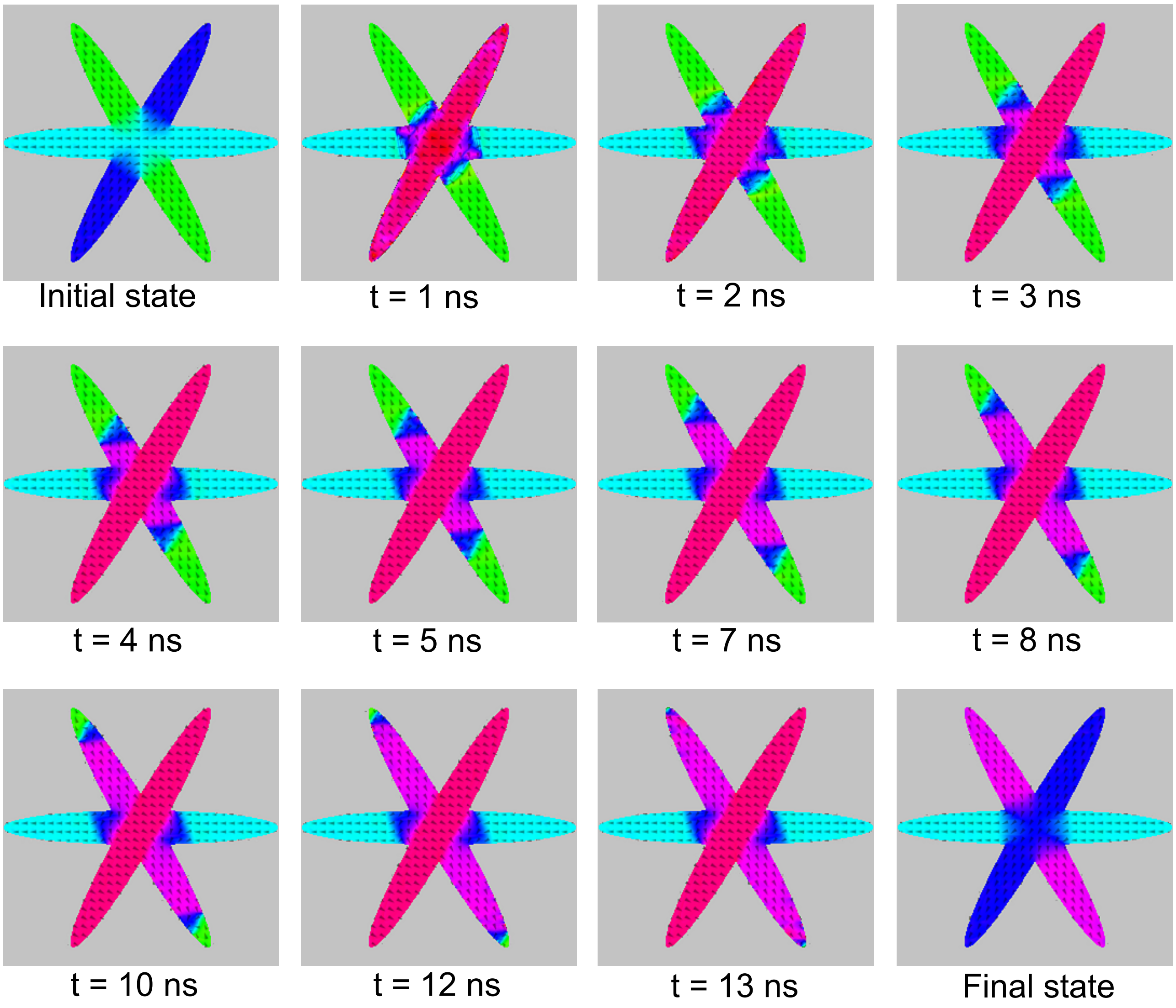

FIG. S2. **Micro-magnetic simulation of switching in Device 2.** Micro-magnetic simulation of a switching from M_1 to M_2 state in Device 2 induced by flowing a spin-polarized current pulse of amplitude 7×10^6 A/cm^2 and duration of 13 ns in the 2-5 arm (see main text). The arrows indicate the magnetization direction.

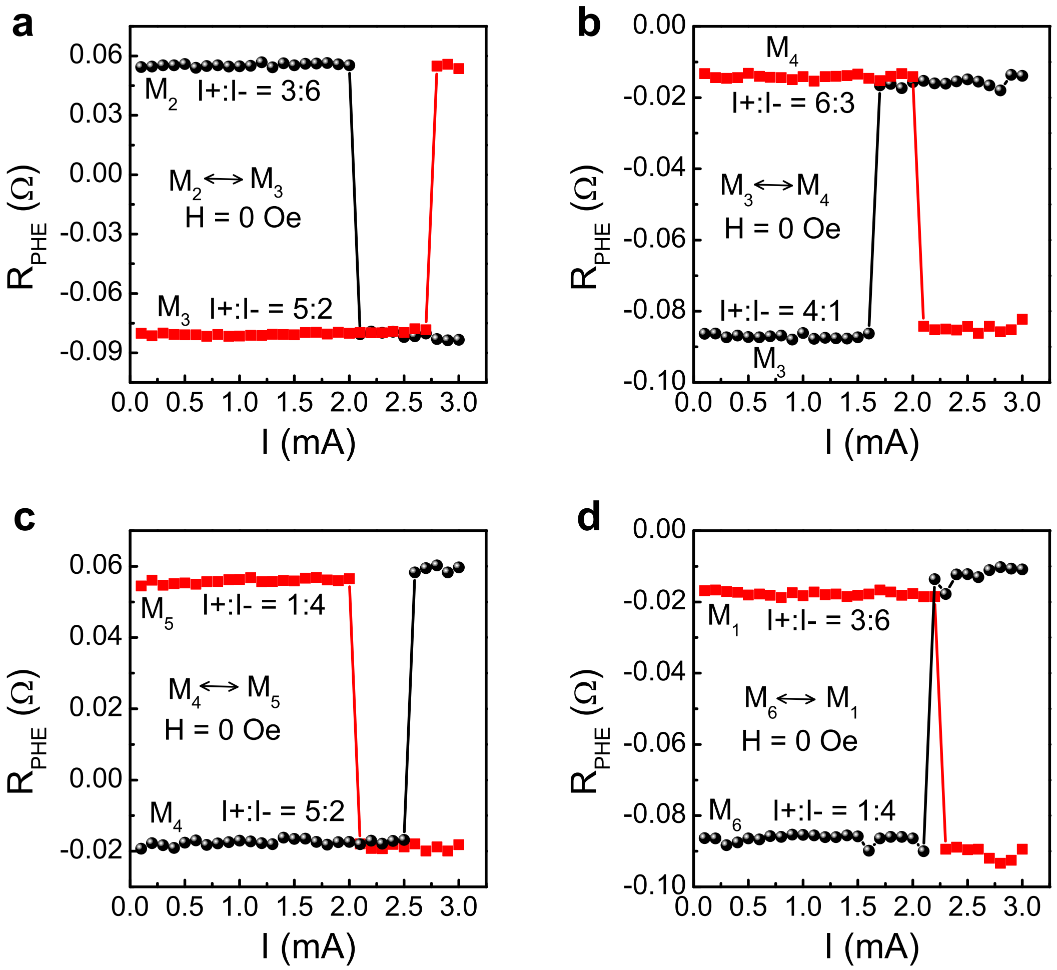

FIG. S3. **SOTs induced switching in Device 2.** (**a**), (**b**), (**c**) and (**d**) Reversible switchings between states M_2 and M_3, M_3 and M_4, M_4 and M_5, M_6 and M_1, respectively, for Device 2.

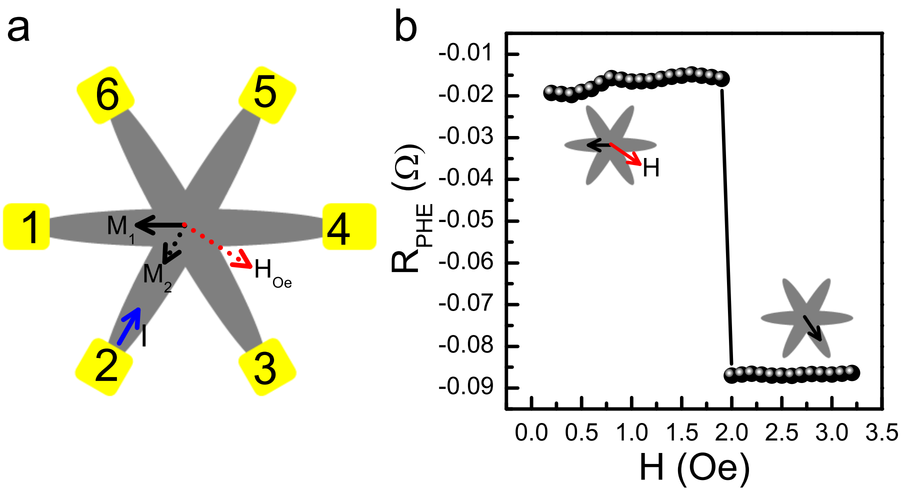

FIG. S4. **SOT mechanism.** (**a**) The schematic diagram indicating the initial state (M_1), final state (M_2), current direction and Oersted field direction. (**b**) The switching from M_1 to M_3 state by applying an external field along $\alpha = 150$ deg.

TABLE I. The switching current magnitude and direction at H = 0 Oe for Device 2.

Final states→ Initial states ↓	M_1	M_2	M_3	M_4	M_5	M_6
M_1		2.5 mA $I_{2\to5}$				2.4 mA $I_{3\to6}$
M_2	2.2 mA $I_{4\to1}$		2.1 mA $I_{3\to6}$			
M_3		2.9 mA $I_{5\to2}$		1.7 mA $I_{4\to1}$		
M_4			2.3 mA $I_{6\to3}$		2.7 mA $I_{5\to2}$	
M_5				2.2 mA $I_{1\to4}$		2.3 mA $I_{6\to3}$
M_6	2.2 mA $I_{1\to4}$				2.8 mA $I_{2\to5}$	

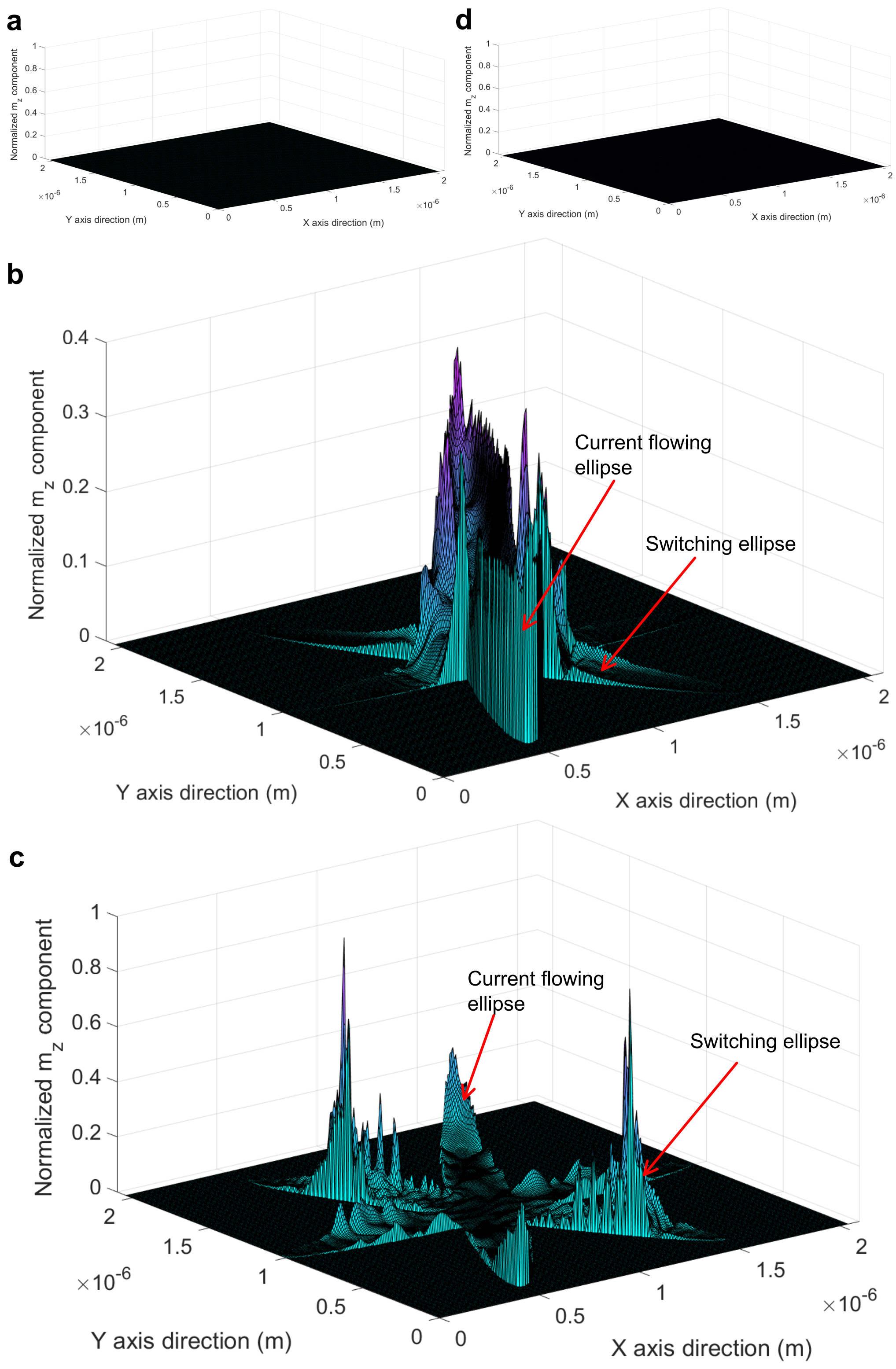

FIG. S5. ***Z*-component of the magnetization during switching.** (**a**) and (**d**) m_Z component in the initial and final states, respectively, of three-crossing ellipses. (**b**) and (**c**) m_Z component at intermediate states of switching.

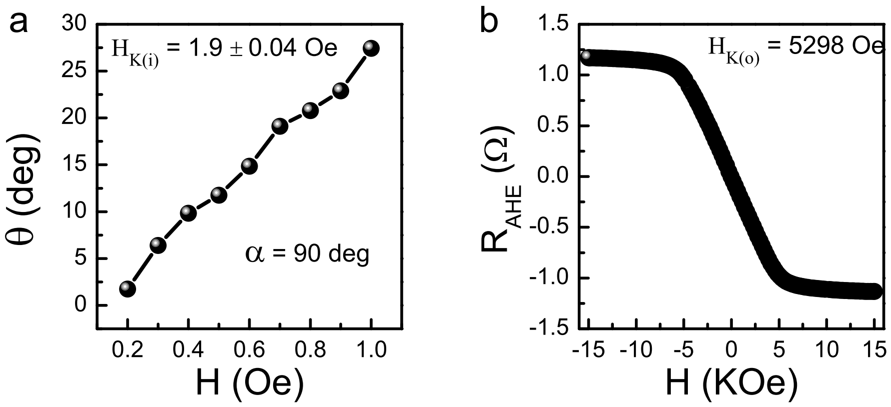

FIG. S6. **Anisotropy fields determination.** (**a**) The change in magnetization direction as a function of a perpendicular in-plane field. (**b**) The change in anomalous Hall resistance as a function of out-of-plane field. The measurements are done with a Hall bar.

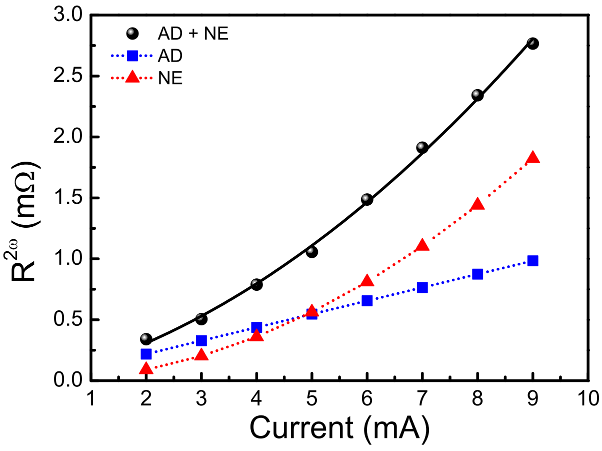

FIG. S7. **Second harmonic Hall measurements.** The cosα contribution to $R^{2\omega}$ as a function of current. The solid black line is a fit using AI + BI^2 and the dotted blue and red lines are AI and BI^2 contributions, respectively.

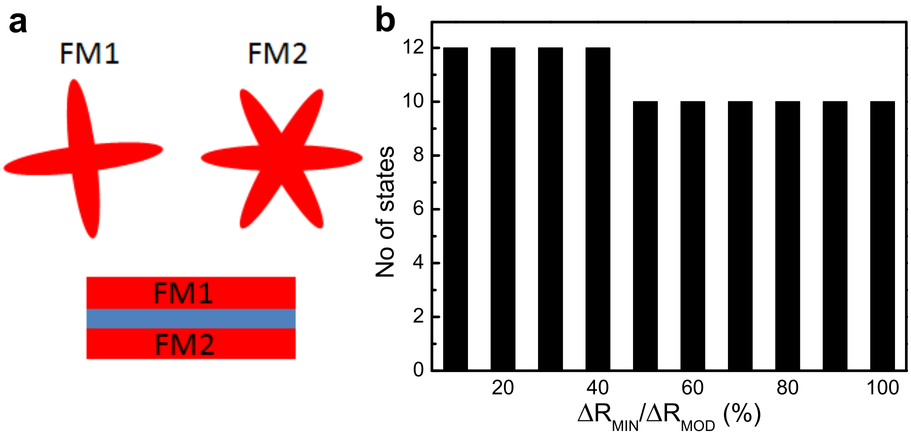

FIG. S8. **12-state MTJ cell.** (**a**) Schematic of a MTJ consisting of two and three crossing ellipses as ferromagnetic layers, where the top layer is rotated by 7 deg. (**b**) Number of simulated distinct resistance states of the MTJ as a function of the smallest difference between the values.

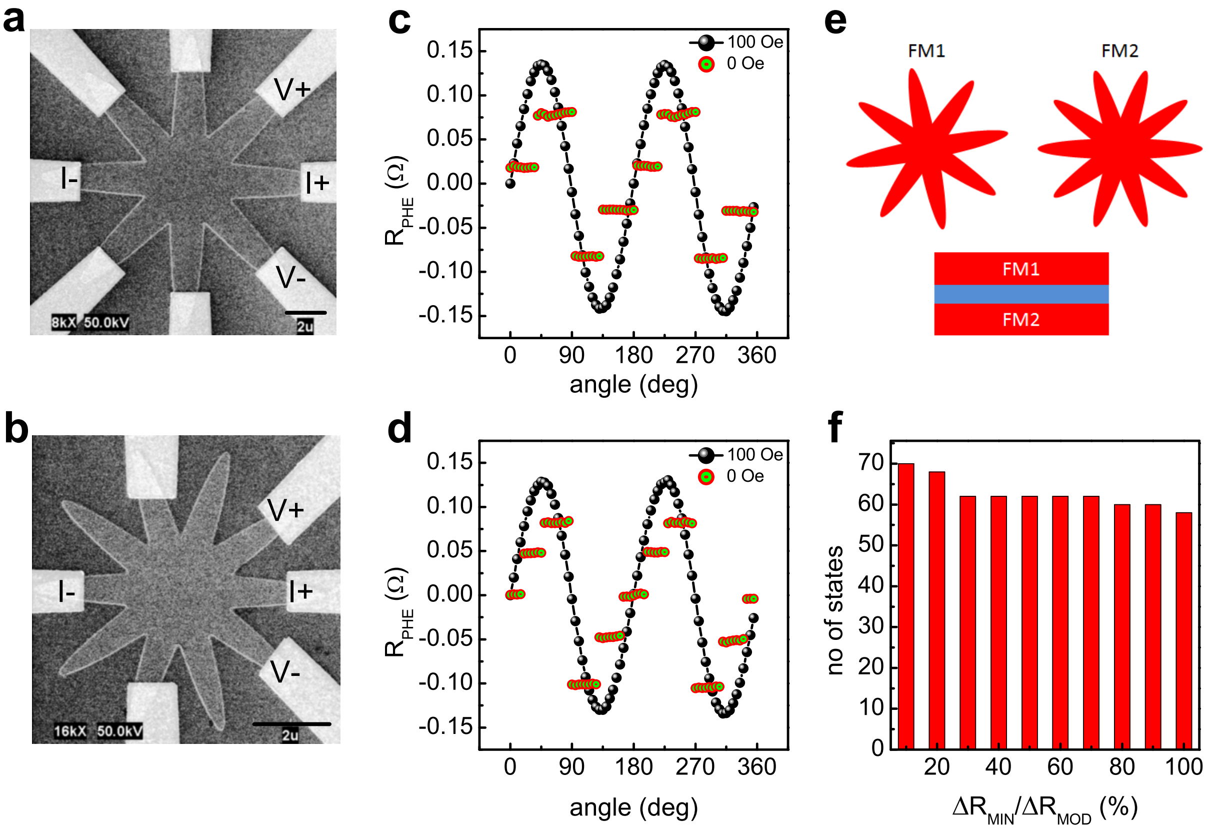

FIG. S9. **70-state MTJ cell.** (**a**) and (**b**) SEM images of four- and five-crossing ellipses, respectively. Current and voltage pads are denoted. (**c**) and (**d**) R_{PHE} is measured at 100 Oe and after the field switched off for each angle α. (**e**) Schematic of a MTJ consisting of four and five crossing ellipses as ferromagnetic layers. (**f**) The simulated number of states of the MTJ as a function of normalized minimum resistance difference between two neighboring states.
